# Effectiveness of Kangaroo Care for a Patient with Postpartum Depression and Comorbid Mother-Infant Bonding Disorder

**DOI:** 10.1155/2019/9157214

**Published:** 2019-02-25

**Authors:** Youji Takubo, Takahiro Nemoto, Yohei Obata, Yoko Baba, Taiju Yamaguchi, Naoyuki Katagiri, Naohisa Tsujino, Toshinori Kitamura, Masafumi Mizuno

**Affiliations:** ^1^Department of Neuropsychiatry, Toho University School of Medicine, 6-11-1 Omori-nishi, Ota-ku, Tokyo 143-8541, Japan; ^2^Department of Psychiatry, Saiseikai Yokohamashi Tobu Hospital, 3-6-1 Shimosueyoshi, Tsurumi-ku, Yokohama, Kanagawa 230-8765, Japan; ^3^Kitamura Institute of Mental Health Tokyo, 2-26-3 Tomigaya, Shibuya-ku, Tokyo 151-0063, Japan; ^4^Kitamura Kokoro Clinic Mental Health, Tokyo, Japan; ^5^T. and F. Kitamura Foundation for Mental Health Research and Skill Advancement, Tokyo, Japan; ^6^Department of Psychiatry, Graduate School of Medicine, Nagoya University, Nagoya, Japan

## Abstract

The presently reported patient was a 37-year-old married primipara with peripartum depression comorbid with bonding disorder. Care anxiety and a lack of affection towards her baby first appeared at around the time of delivery, and the patient developed major depression at one month after the birth of her healthy baby. At first, standard treatment for major depression including the use of antidepressants, electroconvulsive therapy, and supportive psychotherapy were provided. However, bonding problems appeared to impede and obstruct the amelioration of depression. Although treatment methods for bonding disorder have not yet been established, Kangaroo Care was introduced to facilitate skin-to-skin contact. We also educated her in better parenting behavior and provided repeated motivational interviews with her family because a lack of partner and social support and personal temperament (low self-directedness and cooperativeness) were thought to be related to her bonding disorder. This case suggests the effectiveness of Kangaroo Care, which promotes a humanizing maturation of both baby and parent alike, for mothers with postpartum depression and comorbid severe bonding disorder.

## 1. Introduction

Postpartum depression is a common psychological problem that reportedly occurs in as many as 13%–19% of pregnant women worldwide [1]. A Japanese epidemiological survey that rigidly applied the Structured Clinical Interview (SCID) and the Diagnostics and Statistical Manual of Mental Disorders, Third Edition Revised (DSM-ΙΙΙ-R) criteria for the first time in the world, reported an incidence of 5%, with another 5% of pregnant women partially meeting the criteria [2]. Postpartum depression is associated with an increased risk of suicide, decreased maternal sensitivity, and attachment to infants, infanticide, and poor child development [3–5]. Therefore, efforts to detect postnatal depression at an early stage have been increasingly used, including the application of screening tools such as the “Edinburgh Postnatal Depression Scale.” Although the Japanese national fee schedule has recently prioritized high-risk gravidas including those with mental illness, awareness of and care for this condition are not yet sufficient in Japan, especially compared with regional networks of psychiatric treatment facilities and social services in England [6, 7].

Bonding disorder is characterized by aversion to one's infant and a marked impairment in interactions including a lack of parental affective involvement, increased irritability, and infant rejection. The infant's demands can provoke aggressive impulses, leading to verbal abuse and rough treatment when self-control is lost [8]. Parental hostility deprives the infant of a fundamental need for loving relationships, severely impairs interactions, and leads to emotional abuse [9].

The coexistence of postpartum depression and bonding disorder has been previously reported [10–12]. The causal relationships of bonding disorder and maternal depression remain unclear, but a recent large-scale cohort study indicated that bonding disorder predicts depressive moods during pregnancy and 5 days after delivery [13, 14]. The usual treatment for maternal depression does not improve mother-infant relationships [15]. Infants exposed to their mother's hatred and rage may suffer far-ranging and long-term disadvantages, and they have an increased risk of maltreatment [8]. Therefore, when postpartum depression is accompanied by postpartum bonding disorder, treatment should be provided for not only the mother's depressive symptoms, but also the mother-infant relationship.

A standard treatment for bonding disorder has not yet been established. Kangaroo Care is derived from its similarities to marsupial caregiving and was first suggested in Colombia in 1978. The practice was developed as a way of providing compensatory care for low birth weight infants and premature infants [16–18]. Kangaroo Care, also known as skin-to-skin contact, is provided when a diaper-clad infant is placed on the naked chest of a parent [19]. Kangaroo Care is supposed to accelerate autonomic and neurobehavioral maturation in preterm infants [20]. Recent investigations have demonstrated the effectiveness of Kangaroo Care for enhancing bonding with premature infants [21–23]. Therefore, skin-to-skin contact during the postpartum period is recognized as being essential for both the infant's neurological development and the formation of a mother-infant relationship. In the presently reported patient with postpartum depression and bonding disorder, we provided Kangaroo Care accompanied by education regarding the child's communication cues and family sessions. The present case report describes the successful treatment of postpartum depression and comorbid bonding disorder using Kangaroo Care.

## 2. Case Presentation

The patient was a 37-year-old married Japanese woman. She was referred to the Department of Psychiatry at the Toho University Omori Medical Center, Tokyo, to receive care for a severe postpartum depressed mood and intense suicidal ideations. The participants provided written informed consent prior to enrollment in this case report.

She had no previously documented psychiatric history and no documented family history of psychiatric or perinatal illness. She had been brought up in an urban environment since childhood. She had a good relationship with her parents and did not experience any abuse or maltreatment. Her character was honest, diligent, and orderly, and she had an especially strong sense of responsibility. After graduating from university, she worked as an assistant curator in a museum. At the age of 34 years, she met and married her husband, who was an engineer. They were not eager for her to become pregnant. Although she and her husband moved to the countryside because of his work, she decided to continue her job because, despite a 3-hour commute, she found her work to be very fulfilling.

At the age of 36 years, she became pregnant. She quit her job to become a housewife and became bored with her daily life; she also felt that living in the countryside was inconvenient. Her husband was busy with work and left all the pregnancy preparations to her, which caused her to feel frustrated. She started feeling very anxious about her primiparity and child-care, and she moved to her parents' house to receive their support. During her 39th week of pregnancy, she had a forceps delivery because of a birth canal infection. The delivered boy was 3150 grams and had no deformities of any kind. However, she could not hold her child immediately after childbirth because she was receiving treatment for her infection. She appeared to lack affection towards her baby because she could not remember how she felt when she eventually held her baby for the first time.

It was difficult for her to control her baby when he cried, and she felt fatigued and anxious. He was very demanding for his mother's breast milk, and she continued to feed him because of a feeling of responsibility. A month after the delivery, depressive symptoms (depressed mood, abnormal fear, and insomnia) appeared and gradually worsened. She felt a strong sense of distress while she was with her baby and was confused as to how to care for him. She had difficulty asking for help because she felt that she should do everything on her own. She began to regret having given birth because it had led to her present circumstances. Her husband was still unable to provide her with either emotional or physical support, but her mother continued to help her care for her baby. The patient's lack of affection worsened and was accompanied by other depressive symptoms, such as poor concentration, indecisiveness, and reduced energy. The patient began to worsen daily and began to have difficulty taking care of both herself and her baby.

Three months after the birth, she visited a psychiatric outpatient clinic and was diagnosed as having postpartum depression and bonding disorder. Her Temperament and Character Inventory (TCI) scores were as follows: Novelty Seeking (NS): −1.78; Harm Avoidance (HA): 2.52; Reward Dependence (RD): −2.09; Persistence (PS): 1.78; Self-directedness (SD): −2.1; Cooperativeness (CO): −1.35; and Self-transcendence (ST): −0.54. Her temperament was judged as logical and obsessive-compulsive, and her character was judged as melancholic and schizoid.

Treatment with an antidepressant (sertraline, 25 mg/day) and psychotherapy was started, but her depressive symptoms worsened. Because of strong nausea, the treatment with sertraline was discontinued and treatment with mirtazapine (15 mg/day) and olanzapine (initial dose, 2.5 mg/day) was started. Finally, she was admitted to the Department of Psychiatry at the Toho University Omori Medical Center.

On admission, her Hamilton Rating Scale for Depression (HRSD) score was 35. She was characterized as having a depressive mood most of the day, markedly diminished interest and pleasure, insomnia, psychomotor agitation, loss of energy, feelings of worthlessness, indecisiveness, suicidal ideation, and a suspicious attitude. We diagnosed her as having peripartum-onset major depression, and we gradually increased the dosage of mirtazapine up to 45 mg daily and that of olanzapine up to 10 mg daily, with poor results. Therefore, nortriptyline was prescribed along with these drugs. After the dosage of nortriptyline was increased to 100 mg, her depressed mood and indecisiveness began to improve at around day 45 of her hospitalization. She was allowed to stay overnight at her house, with her child, on day 53. However, her depressed mood and anxiety were suddenly exacerbated when she returned to the hospital. Hence, we started a series of electroconvulsive therapy (ECT) treatments beginning on day 72 and her depressive state improved somewhat.

She made comments such as “Even though I should have been happy to have been with my child, it did not go so well emotionally” and “I'm convinced that I'm incurable.” Her score on the Mother to Infant Bonding Scale (MIBS), which is a 10-item self-reported instrument, was 12, indicating a severe bonding disorder [24]. We focused on the bonding disorder and provided Kangaroo Care during family sessions. We started providing Kangaroo Care with her baby for two hours in a private room of the ward while her husband was present. During the Kangaroo Care session, we facilitated skin-to-skin contact and educated her regarding parenting behavior in cooperation with the ward nurses. Two sessions were provided, and similar follow-up care was subsequently provided by midwives. She learned to recognize her baby's gestures, facial expressions, and emotions. She was transfixed and embarrassed when the baby was crying, and she barely smiled at the baby during the first session. To allow the patient to gain self-confidence, we first had the patient hold her baby while he was in a good mood. We repeatedly showed her how to cope with the baby's discomfort in a concrete manner and told her that her baby was very cute to improve her capacity to verbalize her emotions. Her husband was encouraged to send her photos and movies of her child taken at home every day based on the policy of imaginary exposure. After this intervention, her awareness of her child's feelings and her ability to provide flexible care for her baby improved.

**Figure 1:**
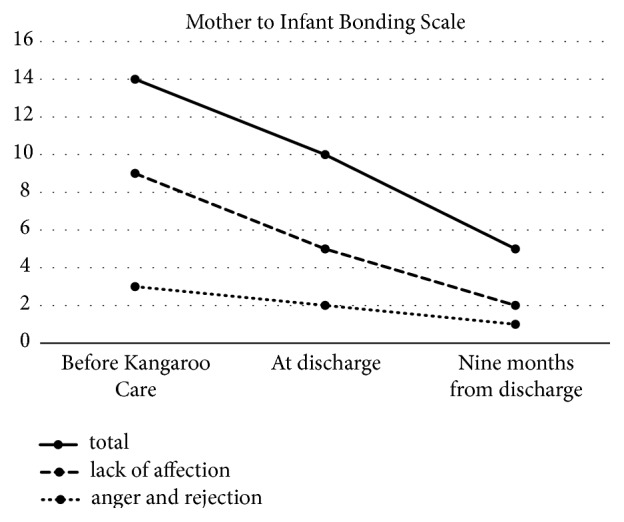


The patient began to experience tender feelings towards her child and her anxiety decreased (HRSD = 7). She tried staying at home overnight, and her depression and bonding problem did not worsen. On day 114, she was discharged from the hospital. She was taking maintenance doses of 100 mg of nortriptyline, 30 mg of mirtazapine, and 5 mg of olanzapine per day at the time of her discharge. We continued outpatient treatment and supported her in caring for her child. In addition, we liaised with midwives, public health nurses, and home visit nurses regarding her care and ideal environment after discharge. At 9 months after her discharge, her MIBS score was 5 (Figure 1). A tendency towards an improvement in her bonding disorder was seen, and her depression remained in remission (HRSD = 6).

## 3. Discussion

The patient met the criteria for major depressive disorder according to the Diagnostics and Statistics Manual of Mental Disorders, 5th ed. (DSM-5), and her depressive symptoms occurred within one month after delivery. Despite the fact that there are no clear diagnostic criteria for bonding disorder, Brockington proposed that bonding disorder was characterized by aversion to infant and a marked impairment in interactions including a lack of affection, increased irritability, and infant rejection [8]. We considered some differential diagnosis (the loss of interest by postpartum depression, the mysophobia of postpartum obsessive-compulsive disorder, reactive attachment disorder, schizoid personality disorder, and so on); however, her bonding problems were not able to be wholly explained by those disorders. Furthermore, she never met the diagnostic criteria of those disorders (except postpartum depression) according to the Diagnostics and Statistics Manual of Mental Disorders, 5th ed. (DSM-5). We observed lack of affection, lack of feelings to protect her child, irritability, and poor parenting styles, and she also realized these symptoms. Her bonding problems corresponded to the Brockington's proposal. Hence, we diagnosed her bonding disorder. In recent Japanese study, Matsunaga performed a two-step cluster analysis using MIBS that suggested an existence of a group of mothers with bonding disorder (14.4% n=104) characterized by severe depression as well as harsher parenting styles that were different from another group (85.6% n=619). In addition, the study showed that the optimal cut-off scores by MIBS were 3/4 at 5 days and 4/5 at 1 month, after childbirth [24]. Her MIBS score at a half year after childbirth was 12. We also asked the same questions as MIBS retrospectively and she looked back that she had had severe bonding problems at 5 days and 1 month after childbirth. Therefore, it is considered that her bonding disorder started right after birth and was severe referred to the MIBS score and interviews. Above all, we diagnosed her as having peripartum-onset major depressive disorder and bonding disorder. The causal relationship between bonding disorder and postpartum depression is unclear [13]. In the present case, a lack of affection towards her baby preceded her depressive state. Therefore, the bonding disorder was thought to have led to the patient's depression in the present case.

The causes of postpartum bonding disorder are multifaceted. Support from the woman's partner and social support during pregnancy and the postpartum period are significantly correlated with bonding disorder [25–27]. We repeatedly conducted motivational interviews with her husband and family to strengthen her support system by clarifying the roles of her extended family. From the viewpoint of temperament and character, which are included in the TCI, a low SD and CO might be correlated with bonding disorder [28]. We suggested to her family that these characteristics of hers might be correlated with her bonding problem. In addition, attitudes towards pregnancy, emotions during labor, and perceived parental care and control during childhood are known to be correlated with each other [13, 29, 30]. Two factors in the structure of the MIBS have been found: “anger and rejection” and “lack of affection” [31]. “Lack of affection” was predominant in her bonding disorder. Little is known about the difference in the psychosocial background between a “lack of affection” predominant group and an “anger or rejection” predominant group. However, it is suggested that low SD and CO, poor partner and social support, and negative attitudes towards pregnancy elevated “lack of affection.”

Although the prevention and treatment of bonding disorder have not yet been established, Brockington pointed out that such care should be focused on promoting mother-infant interactions [8]. Muzik proposed that bonding impairment and observed parenting behaviors affect flexibility, engagement, warmth, and sensitivity when caring for a baby, and these factors are significantly correlated with each other [25]. Bonding disorder can lead to problems associated with the mother's parenting behavior and the child's unsettled condition, and the child's condition can lead to a reduction in the mother's self-esteem and affect bonding adversely. To neutralize this negative chain reaction, Kangaroo Care appears to be indispensable as a treatment that focuses on the mother-infant relationship.

Regarding the definition of Kangaroo Care, a practical guide by World Health Organization (WHO) proposed its key features: early, continuous, and prolonged skin-to-skin contact between the mother and the baby, exclusive breastfeeding, initiated in hospital and continued at home, adequate support, follow-up in outpatient settings, and so on. [32]. Systematic review shows there was various definitions. Although a large number of studies did not describe the definition of Kangaroo Care, 71% of studies included skin-to-skin contact. And, a period of time (undefined [37%], continuous within one session [39%], and 24 hours per day [14%]), duration (undefined [65%], one or two sessions [31%]), and monitoring by an experienced nurse were in various ways among the studies [33, 34]. Therefore, it is thought that the standard method of Kangaroo Care has not been established yet. Moreover, subjects were infants with low birth weight and their mother in most of the studies. In the present case, the care is characterized by a combination with assertive psychological interventions that included education of parenting behaviors. The time point initiated the care was also different from other studies. In many cases, Kangaroo Care was provided right after birth, but our care was provided a half year after birth.

Kangaroo Care promotes a humanizing maturation of both baby and parent alike. Psychiatric mother-and-baby units for providing Kangaroo Care have recently become quite common in British National Health Services areas [6]. However, few reports have been published regarding the effectiveness of Kangaroo Care for mothers with depression accompanied by bonding disorder. In the present case, the standard treatments for depression including antidepressants, ECT, and supportive psychotherapy were only somewhat effective at alleviating the patient's depression, and her bonding problems appeared to impede and obstruct the amelioration of her depression. The introduction of Kangaroo Care and psychoeducation for her family seemed to improve her bonding disorder, which led to the remission of her depression. This case indicates the effectiveness of Kangaroo Care for mothers with postpartum depression and comorbid severe bonding disorder.

Bonding disorder may improve naturally, and knowledge about the trajectory of the severity of bonding disorder remains insufficient [35]. The patient's parenting behaviors remarkably improved after two sessions and she recovered self-confidence. Better parenting behaviors and self-confidence seems to be the base for improving bonding disorder in outpatient settings. Although Kangaroo Care does not seem to be different between inpatient and outpatient care settings methodologically, we believe intensive intervention in inpatient care can maintain its effectiveness.

Further reports and studies are needed to clarify the diagnostic criteria for bonding disorder, the definition of Kangaroo Care, and the effectiveness of Kangaroo Care in mothers with postpartum depression and comorbid bonding disorder.

## 4. Conclusion

This case suggests the effectiveness of Kangaroo Care, which promotes a humanizing maturation of both mother and infant, for mothers with postpartum depression and comorbid severe bonding disorder.

## References

[1] O'Hara M. W., McCabe J. E. (2013). Postpartum depression: current status and future directions. *Annual Review of Clinical Psychology*.

[2] Kitamura T., Yoshida K., Okano T. (2006). Multicentre prospective study of perinatal depression in Japan: incidence and correlates of antenatal and postnatal depression. *Archives of Women's Mental Health*.

[3] Lindahl V., Pearson J. L., Colpe L. (2005). Prevalence of suicidality during pregnancy and the postpartum. *Archives of Women's Mental Health*.

[4] Paulson J. F., Dauber S., Leiferman J. A. (2006). Individual and combined effects of postpartum depression in mothers and fathers on parenting behavior. *Pediatrics*.

[5] Marmorstein N. R., Malone S. M., Iacono W. G. (2004). Psychiatric disorders among offspring of depressed mothers: associations with paternal psychopathology. *The American Journal of Psychiatry*.

[6] Elkin A., Gilburt H., Slade M. (2009). A national survey of psychiatric mother and baby units in England. *Psychiatric Services*.

[7] Okano T. (2014). Comparison of care for perinatal mental health between Japan and other countries. *The Japanese Society of Psychiatry and Neurology*.

[8] Brockington I. (2011). Maternal rejection of the young child: present status of the clinical syndrome. *Psychopathology*.

[9] Ohashi Y., Sakanashi K., Tanaka T., Kitamura T. (2016). Mother-To-Infant bonding disorder, but not depression, 5 days after delivery is a risk factor for neonate emotional abuse: a study in Japanese mothers of 1-month olds. *The Open Family Studies Journal*.

[10] Edhborg M., Matthiesen A.-S., Lundh W., Widström A.-M. (2005). Some early indicators for depressive symptoms and bonding 2 months postpartum—a study of new mothers and fathers. *Archives of Women's Mental Health*.

[11] Klier C. M. (2006). Mother-infant bonding disorders in patients with postnatal depression: the postpartum bonding questionnaire in clinical practice. *Archives of Women's Mental Health*.

[12] Moehler E., Brunner R., Wiebel A., Reck C., Resch F. (2006). Maternal depressive symptoms in the postnatal period are associated with long-term impairment of mother-child bonding. *Archives of Women's Mental Health*.

[13] Kokubu M., Okano A., Sugiyama T. (2012). Postnatal depression, maternal bonding failure, and negative attitudes towards pregnancy: a longitudinal study of pregnant women in Japan. *Archives of Women's Mental Health*.

[14] Ohara M., Okada T., Kubota C. (2017). Relationship between maternal depression and bonding failure: a prospective cohort study of pregnant women. *Psychiatry and Clinical Neurosciences*.

[15] Forman D. R., O'Hara M. W., Stuart S., Gorman L. L., Larsen K. E., Coy K. C. (2007). Effective treatment for postpartum depression is not sufficient to improve the developing mother-child relationship. *Development and Psychopathology*.

[16] Charpak N., Ruiz-Peláez J. G., De Calume Z. F. (1996). Current knowledge of kangaroo mother intervention. *Current Opinion in Pediatrics*.

[17] Doyle L. W. (1997). Kangaroo mother care. *The Lancet*.

[18] Charpak N., Ruiz-Peláez J. G., de Calume F., Charpak Y. (1997). Kangaroo mother versus traditional care for newborn infants ≤2000 grams: a randomized, controlled trial. *Pediatrics*.

[19] Kommers D. R., Joshi R., van Pul C. (2017). Features of heart rate variability capture regulatory changes during kangaroo care in preterm infants. *Journal of Pediatrics*.

[20] Feldman R., Eidelman A. I. (2003). Skin-to-skin contact (Kangaroo Care) accelerates autonomic and neurobehavioural maturation in preterm infants. *Developmental Medicine & Child Neurology*.

[21] Brett J., Staniszewska S., Newburn M., Jones N., Taylor L. (2011). A systematic mapping review of effective interventions for communicating with, supporting and providing information to parents of preterm infants. *BMJ Open*.

[22] Evans T., Whittingham K., Sanders M., Colditz P., Boyd R. N. (2014). Are parenting interventions effective in improving the relationship between mothers and their preterm infants?. *Infant Behavior & Development*.

[23] McGregor J., Casey J. (2012). Enhancing parent-infant bonding using kangaroo care: a structured review. *Evidence Based Midwifery*.

[24] Matsunaga A., Takauma F., Tada K., Kitamura T. (2017). Discrete category of mother-to-infant bonding disorder and its identification by the mother-to-infant bonding scale: a study in Japanese mothers of a 1-month-old. *Early Human Development*.

[25] Muzik M., Bocknek E. L., Broderick A. (2013). Mother-infant bonding impairment across the first 6 months postpartum: the primacy of psychopathology in women with childhood abuse and neglect histories. *Archives of Women's Mental Health*.

[26] Margison F., Brockington I. (1982). *Psychiatric Mother and Baby Units. Motherhood and Mental Illness*.

[27] Ohara M., Okada T., Aleksic B. (2017). Social support helps protect against perinatal bonding failure and depression among mothers: a prospective cohort study. *Scientific Reports*.

[28] Ohashi Y., Kitamura T., Kita S., Haruna M., Sakanashi K., Tanaka T. (2014). Mothers bonding attitudes towards infants: impact of demographics, psychological attributes, and satisfaction with usual clinical care during pregnancy. *International Journal of Nursing and Health Science*.

[29] Grant K. A., Bautovich A., McMahon C., Reilly N., Leader L., Austin M. P. (2012). Parental care and control during childhood: associations with maternal perinatal mood disturbance and parenting stress. *Archives of Women's Mental Health*.

[30] Weisman O., Granat A., Gilboa-Schechtman E. (2010). The experience of labor, maternal perception of the infant, and the mother's postpartum mood in a low-risk community cohort. *Archives of Women's Mental Health*.

[31] Yoshida K., Yamashita H., Conroy S., Marks M., Kumar C. (2012). A Japanese version of mother-to-infant bonding scale: Factor structure, longitudinal changes and links with maternal mood during the early postnatal period in Japanese mothers. *Archives of Women's Mental Health*.

[32] (2003). *Kangaroo Mother Care: A Practical Guide*.

[33] Chan G. J., Valsangkar B., Kajeepeta S., Boundy E. O., Wall S. (2016). What is kangaroo mother care? Systematic review of the literature. *Journal of Global Health*.

[34] Ghavane S., Murki S., Subramanian S., Gaddam P., Kandraju H., Thumalla S. (2012). Kangaroo mother care in kangaroo ward for improving the growth and breastfeeding outcomes when reaching term gestational age in very low birth weight infants. *Acta Paediatrica*.

[35] Van Bussel J. C. H., Spitz B., Demyttenaere K. (2010). Three self-report questionnaires of the early mother-to-infant bond: Reliability and validity of the Dutch version of the MPAS, PBQ and MIBS. *Archives of Women's Mental Health*.

